# An inquiry into labor conditions across key rural sectors in Africa

**DOI:** 10.1371/journal.pone.0338694

**Published:** 2025-12-22

**Authors:** Jackson Elias Nzira, Martin C. Parlasca, Matin Qaim

**Affiliations:** 1 Center for Development Research (ZEF), University of Bonn, Bonn, Germany; 2 Institute for Food and Resource Economics, University of Bonn, Bonn, Germany; Università degli Studi di Milano: Universita degli Studi di Milano, ITALY

## Abstract

While agriculture remains a key source of livelihoods in rural Africa, employment in other economic sectors is gaining in importance. However, details of the labor conditions are under-researched. Here, we examine labor conditions in different sectors of the rural economy using survey data from wage workers and key employers in Kenya, Namibia, Tanzania, and Zambia. We find that close to 60% of the adult population are self-employed in agriculture or small non-agricultural businesses, whereas only 7% are wage-employed. Over 60% of those in wage employment earn below the minimum wage. The main employers are agricultural farms and small private firms in hospitality and other services sectors. Average working conditions remain poor. Payment above the minimum wage, employment stability, and certain social benefits are more widely observed in sectors such as public administration, education, and healthcare, where longer-term or permanent contracts are common. Workers in agriculture and construction predominantly depend on seasonal and temporary contracts. Although individual education levels, training, and experience enhance payment and job quality, those with higher education often report lower job satisfaction. Our findings underscore the need for policies that broaden wage employment opportunities and improve the labor conditions in rural Africa.

## 1. Introduction

Subsistence agriculture has traditionally served as the primary livelihood for most rural households in sub-Saharan Africa, sometimes combined with different forms of self-employment such as gathering and selling environmental goods, petty trading, artisanal crafts, or food vending. However, the role of wage employment has grown over time for rural households to augment and stabilize their incomes [1–6]. This gradual shift from subsistence livelihoods to more diversified and wage-dependent income sources reflects broader rural transformation and structural change, as economies move from agriculture-centered systems toward more varied and productivity-focused rural economies [7–10].

Due to continued rural population growth, limited farmland availability, and increasing risk of farm production due to climate change, the need for rural wage employment, also outside of the agricultural sector, will likely further grow in the future [11–14]. Understanding how the expansion of wage employment influences inclusive and sustainable rural transformation is vital for policymakers and development practitioners. It is essential to assess whether these rural jobs provide productive, fair wages, maintain safe working environments, and include sufficient social protection.

However, wage employment in rural Africa has, so far, received relatively little attention. While there is a substantial body of research examining the role of off-farm employment, much of this literature approaches the topic in a highly aggregated manner, without delving into the specific types of off-farm employment, differences in working conditions, or variations across sectors [14–18]. In this article, we address this research gap, pursuing three main objectives. First, we analyze the prevalence of wage employment in rural Africa, distinguishing between different sectors and types of employers. Second, we assess job satisfaction of employees and their working conditions, which include wages, but also contract types, occupational safety, and other types of benefits. Third, we investigate which employer and employee characteristics determine job satisfaction and working conditions in rural Africa. A better understanding of these issues is critical for developing targeted strategies to increase the availability of employment opportunities and improve the working conditions for rural households.

We use data from two surveys, one conducted with male and female adults (N = 6,722) and the other with employers (N = 610) in rural regions of four African countries, namely Kenya, Namibia, Tanzania, and Zambia. This approach of combining employee and employer data across countries and regions facilitates a deeper understanding of rural job situations. What should be noted is that our data involves samples that are not representative at the national level for any of the countries studied. This means that the findings cannot be used for country-level claims or comparisons. Nevertheless, the general patterns we observe regarding labor conditions across heterogenous rural settings in sub-Saharan Africa provide broader and valuable insights into sectoral employment dynamics.

We add to the existing literature in two main ways. First, most studies on off-farm employment examine the role of wage income from a household perspective but lack nuance in terms of different employment sectors. A few case studies on wage employment conditions in specific agricultural value chains exist [18–24]. But we are not aware of studies on labor conditions in non-agricultural rural sectors, such as construction, transport, retailing, or tourism. Second, we are not aware of other research that combines survey data from employers and employees to comprehensively understand the labor conditions in rural Africa. By breaking down rural wage employment by sector and employer type, and directly surveying both employers and workers, we assess working conditions in agricultural and non-agricultural sectors in rural sub-Saharan Africa. This broad sectoral approach is essential because rural labor markets are becoming more diverse. Yet, policies often focus mainly on agricultural wage labor, possibly neglecting employment trends and working conditions in the expanding rural non-farm economy.

The remainder of this article is organized as follows: In Section 2, we present the materials and methods, including an overview of the study areas across the four countries, the sampling frameworks, data collection procedures, and statistical methods of data analysis. In Section 3, we present and discuss our findings. Finally, in Section 4, we conclude and discuss policy implications emerging from our results.

## 2. Materials and methods

### 2.1. Ethical statement

The Ethics Board of the Center for Development Research (ZEF), University of Bonn, granted ethical approval for this study. Necessary research clearances were obtained from relevant national and local authorities in each study area. In Kenya, permission was granted by the National Commission for Science, Technology and Innovation (NACOSTI) and local authorities in Baringo County. In Tanzania, approvals came from the Tanzania Commission for Science and Technology (COSTECH) and regional authorities in Morogoro and Iringa. Namibia’s study received authorization from the National Commission on Research, Science and Technology (NCRST) and the Zambezi regional government. In Zambia, the University of Zambia’s Directorate of Research and Graduate Studies provided ethical approval, with additional research permits secured from the National Science and Technology Council (NSTC) and the Western Province administration. In all sites, local leaders at ward, village, and community levels gave consent before data collection.

### 2.2. Study areas

We use primary data collected from rural areas in Baringo County (Kenya), the Zambezi Region (Namibia), Morogoro and Iringa Regions (Tanzania), and the Western Province (Zambia). These study areas were intentionally selected for their different climatic, agro-ecological, economic, social, cultural, and institutional characteristics. While these regions are not fully representative for each of the study countries, together, they represent a broad spectrum of conditions typical of rural sub-Saharan Africa. Further details are provided below.

***Kenya.*** Baringo County, one of Kenya’s poorest regions, was selected for its role in thermal energy and the Lamu Port South Sudan-Ethiopia Transport (LAPSSET) infrastructure projects. Baringo has a low population density and high poverty rate, reflecting significant economic challenges [25]. The local economy revolves around livestock, especially ruminants, with some farming of maize, beans, and vegetables, even though frequent droughts limit crop productivity. Thermal energy projects, particularly geothermal exploration, have created jobs in manual labor and security for low-skilled workers. Community conservancies provide opportunities for tourism-based wage employment, but poor infrastructure, limited electricity, and banditry hinder its full potential.

***Namibia.*** The Zambezi Region, one of Namibia’s poorest, is located within the Kavango-Zambezi Transfrontier Conservation Area (KAZA TFCA), a vital wildlife corridor in southern Africa. Agriculture, including cattle, goats, and maize, is a primary livelihood, but high unemployment persists due to severe droughts and recurring floods. The Namibian government provides cash transfers to support families impacted by these challenges. Nature conservancies and national parks offer limited job opportunities in tourism, including hospitality, tour guiding, and administration.

***Tanzania.*** The regions of Morogoro and Iringa in Tanzania were chosen for their strategic location within the Southern Agricultural Growth Corridor (SAGCOT)—a program aimed at supporting small businesses in the agricultural sector, improving protection against land grabbing, creating jobs, and enhancing infrastructure and food security. Morogoro is the largest rice-producing region in the country, while Iringa leads in vegetable production and is a major producer of maize, beans, and potatoes. Many households engage in food processing, such as producing flour and sunflower oil. Both regions have public and private forest reserves that provide resources like charcoal, firewood, and timber [26].

***Zambia.*** The Western Province of Zambia borders Namibia’s Zambezi region to the south and is part of the KAZA TFCA, which includes vital private and communal conservancies for wildlife conservation and tourism. Despite its rich natural resources, Western Province remains one of Zambia’s poorest regions, with a poverty rate of 79% [27]. Many households depend on small-scale farming, growing maize, cassava, and groundnuts, alongside livestock husbandry. Wildlife conflicts often result in crop and livestock losses, while other key economic activities include tourism, artisanal fishing, and forest product harvesting.

### 2.3. Survey sampling

Our analysis focuses on adult individuals of rural households and on employers in all relevant sectors within the study areas. Households for the survey were selected through a two-stage stratified random sampling process across all four countries. Initially, enumeration areas (EAs) in the selected regions were identified based on population strata, with a set number of EAs randomly chosen for each stratum. Subsequently, households were randomly selected within the chosen EAs. In each household, we collected household-level data as well as data for adult individuals, covering 703 households in Kenya’s Baringo County (1,717 individuals), 652 households in Namibia’s Zambezi region (1,765 individuals), 870 households in Tanzania’s Iringa and Morogoro regions (2,086 individuals), and 437 households in Zambia’s Western Province (1,154 individuals).

For the employer survey, a purposive sampling method was used to select relevant employers from rural sectors across the same regions where the household survey was conducted. An employer, in this context, refers to an individual or entity that hires one or more individuals in exchange for wages or salaries. A purposive sampling approach was chosen due to the lack of comprehensive lists of all rural employers and to ensure that all relevant employment sectors are covered. In total, we selected 136 employers in Kenya, 140 in Namibia, 220 in Tanzania, and 114 in Zambia.

### 2.4. Data collection

Data collection for both the household and the employer surveys took place between May and August 2023, using structured questionnaires. All participants provided verbal informed consent after being informed of their rights and the study’s purpose, procedures, and intended use of data. Enumerators documented verbal consent by recording participants’ agreement in the survey instruments before beginning data collection. Local enumerators, trained and supervised by the researchers, utilized computer-assisted personal interviewing techniques to gather the information. The questionnaires were carefully pre-tested to ensure relevance and clarity. The household survey captured household characteristics, economic activities, wealth, access to infrastructure and institutions, and individual labor conditions.

For the employer survey, we interviewed three categories of employers: (1) enterprises, (2) public institutions, and (3) NGOs. In this context, enterprises refer to private-sector entities, covering all types of businesses (including farms) that operate for profit and hire individuals for income-generating activities. Public institutions include government agencies and publicly funded organizations that provide employment as part of their public service mandates. NGOs refer to non-governmental, nonprofit organizations that hire personnel to fulfill their missions. The employer data collected includes information on ownership structures, business registration status, number of employees, sectors of operation, prospects for expansion, challenges for growth, wage structures, working hours, and other relevant indicators of labor conditions. The interviews were primarily conducted with the owners or managers of the organization; however, if they were unavailable, other knowledgeable management personnel were interviewed.

### 2.5. Measuring labor conditions

We start the analysis using descriptive statistics to characterize rural employers and employees across the various economic sectors and study areas. Then, based on household- and individual-level data, we analyze labor conditions along four key dimensions: (1) wages, (2) type of job contracts, (3) decent work index, and (4) job satisfaction. To make wages comparable, we calculate the hourly wage of individuals based on the cash payment received, the frequency of payment, and the number of hours worked. Most workers receive a fixed cash wage, which is paid with varying frequency, depending on the activity and the type of employment contract—such as permanent, temporary, seasonal, or no contract. Wage is a fundamental aspect of job quality [28,29] and is therefore assessed in addition to other non-wage aspects.

Beyond wages and contractual arrangements, we evaluate job quality using the concept of decent work as defined by the International Labor Organization (ILO). Decent work refers to employment that is productive and fairly paid, providing security in the workplace, social protection for families, and equal opportunities for all workers. To implement this concept at the individual level, we construct a decent work index (DWI), following Fabry et al. [24]. We consider monetary and non-monetary aspects of employment. Non-monetary factors, such as fringe benefits and non-wage elements (e.g., working time, occupational safety, health insurance, paid holidays, maternity/paternity leave), importantly contribute to job value and quality. Building on International Labor Organization guidelines [30], our DWI focuses on sector-specific dimensions relevant to individual workers. It is designed to be measurable at the individual level and employs objective indicators to minimize potential self-reporting bias [31].

More specifically, we construct a DWI based on the following indicators: (1) adequate earnings and productive work, (2) decent work time, (3) stability and security of work, (4) safe work environment, and (5) social protection. The first indicator assesses whether workers receive the national minimum wage. Minimum wage thresholds per hour of work are specific to each country: 75 KSH for Kenya, 18 NAD for Namibia, 718 TZS for Tanzania, and 7.15 ZMW for Zambia. Fringe benefits (e.g., transportation, housing, meals) and training provided by employers are also considered. To facilitate comparisons, we express hourly wages in purchasing power parity (PPP) dollars, using World Bank PPP exchange rates for 2023.

The second DWI indicator assesses whether the individual’s weekly work hours with a particular employer are capped at 48 hours, with adequate compensation provided for any hours exceeding this limit. Furthermore, it examines whether the worker has to work during night-time and/or public holidays, whether such work times are appropriately compensated, and whether paid leave is provided. The third indicator evaluates the stability and security of work, examining factors related to the continuity and predictability of employment, by analyzing workers’ contract types and durations. The fourth indicator assesses conditions related to occupational safety by examining whether the work involves the handling of dangerous products (e.g., pesticides, other toxic materials) without protection or other activities with high risks of accidents. The last DWI indicator assesses whether the worker is entitled to social benefits, including health insurance coverage, sick leave, and maternity/paternity leave.

For each DWI indicator, we compute a score value by averaging the dimensions specific to that indicator. Subsequently, we calculate the overall DWI for an individual by averaging the score values for the five indicators [24]. The DWI ranges between 0 and 1, with higher values indicating better working conditions (for more details, see Table A in S1 File). We compare the mean DWI across sectors to identify possible differences.

To validate our decent work index, we perform two tests. First, we calculate Cronbach’s alpha to determine whether the five components are related in a meaningful way and can be combined into a single index. The results show acceptable reliability for our five-item index. Second, we conduct principal component analysis to see whether the five components represent a common underlying dimension of job quality or are unrelated aspects. The analysis confirms that all five components load positively on the first principal component and collectively explain a significant proportion of variance, supporting the use of a composite index (detailed validation results and discussions of methodological considerations are provided in the S1 File).

Beyond objectively quantifiable indicators, subjectively perceived factors of the individual employment situation can also be important for people’s wellbeing. In the survey, we asked employed individuals for their personal job satisfaction with three response options, namely “dissatisfied”, “indifferent”, and “satisfied”. These responses are also used for the statistical analysis of labor conditions.

### 2.6. Analyzing determinants of labor conditions

We use regression models to analyze factors that influence the labor conditions in rural Africa, using the household- and individual-level data as well as the data from the employer survey across the four study countries. For the models with individual-level data, we consider three outcome variables – hourly wages, DWI, and job satisfaction – and regress them on household and individual characteristics, dummies for the sector in which the individual is employed, and other employer-related factors. In line with human capital theory [32,33], we consider education and experience as essential factors influencing wages. Additionally, efficiency wage models [34] support our inclusion of employer characteristics and sectoral differences that impact how wages are set. The theory of compensating differentials [35] also indicates that wages, working conditions, and job satisfaction are interconnected and vary across sectors. For the models with employer-level data, we estimate the extent to which wages paid are associated with employer characteristics, including size of the organization/entity, ownership, registration status, years in operation, and trade union affiliation, among others.

More specifically, we estimate regression models of the following type with individual-level data:


$$\;\;\;\;\;\;\;\;\;\;\;\;\;\;\;\;\;\;\;\;\;\;\;\;\;\;\;\;\;\;\;Y_{ij}=\beta_{\text{0}}+\beta_{\text{1}}{Sec}_{ij}+\beta_{\text{2}}{Ind}_{ij}+\beta_{\text{3}}{Emp}_{ij}+\gamma C_{j}+\varepsilon_{ij}$$ (1)

where $\;Y_{ij}$ is the outcome variable (hourly wage, DWI, job satisfaction) for individual i in household j; ${Sec}_{ij}$ represents the sector in which individual i is employed, ${Ind}_{ij}$ is a vector of individual characteristics (e.g., sex, age, level of education, experience), ${Emp}_{ij}$ includes job-related characteristics, such as where the job is located (e.g., village, town) and whether or not specific in-job training was received, and $\varepsilon_{ij}$ is the error term. As we pool the data across study countries, we also include a vector of country fixed effects, $C_{j}$.

For wages and DWI as continuous outcome variables, we use ordinary least squares (OLS) to estimate eq (1). Job satisfaction is measured as a categorical variable with three response options, so we use an ordered logit estimator. In the job satisfaction model, we additionally include DWI as an explanatory variable to test whether objectively measurable work conditions correlate with subjectively-felt job satisfaction.

To analyze wage rates with the employer survey data, we estimate the following model:


$$\;\;\;\;\;\;\;\;\;\;\;\;\;\;\;\;\;\;\;\;\;\;\;\;\;\;\;\;\;\;\;\;\;\;\;\;\;\;\;\;\;\;\;\;Z_{k}=\alpha_{\text{0}}+\alpha_{\text{1}}{Sec}_{k}+\alpha_{\text{2}}{Empch}_{k}+\gamma C_{k}+\mu_{k}$$ (2)

where $Z_{k}$ is the average hourly wage paid by employer *k*, ${Sec}_{k}\;$is a vector of dummies representing the economic sector in which employer *k* operates, ${Empch}_{k}\;$ is a vector of other employer characteristics (e.g., size of the organization, ownership status, etc.), and $\mu_{k}$ is a random error term. Again, we include a vector of country fixed effects, $C_{k}$.

### 2.7. Robustness check

To ensure the reliability of our findings, we conduct several robustness checks using household survey data on wage employees, with all results reported in the S1 File. First, we re-estimate our main models using alternative country groupings by pooling data from the study regions in Tanzania and Kenya, and separately pooling data from the study regions in Namibia and Zambia (Table I in S1 File). Second, to examine whether the determinants of wages and decent work conditions vary across the distribution of these outcomes, we employ quantile regression at the 25th, 50th, and 75th percentiles for both hourly wages and the decent work index (Tables J and K in S1 File). This approach is especially useful because mean-based estimates can mask heterogeneity across workers at different points in the wage and job quality distributions [36].

Third, we use an alternative measure of decent work—decent work incidence—by constructing a binary variable differentiating between workers whose decent work index falls above (value of one) or below (value of zero) a threshold defined as the median minus one standard deviation. This approach follows established methods in poverty and deprivation measurement, such as the multidimensional poverty index [37]. It offers a complementary perspective on job quality by using workers in particularly precarious employment situations as the reference that should be improved (Table K in S1 File). Fourth, we disaggregate the decent work index by estimating separate OLS regressions for each of its five components to determine which specific dimensions of job quality are most strongly linked to employer and employee characteristics (Table L in S1 File). These robustness checks consistently support our main findings.

## 3. Results and discussion

### 3.1. Characteristics of rural employers

Table 1 presents key characteristics of the employers surveyed across the study areas and countries (a distribution by economic sector is shown in Table C in S1 File). In all four countries, over 60% of the employers are private enterprises (including farms and informal businesses in other sectors), followed by public institutions. NGOs as rural employers are less often observed in the study regions. Of the enterprises, the large majority are owned by locals, though with some differences across the countries. In the study region in Tanzania, 19% of enterprises are owned by people from other regions within the country, whereas in Zambia, 22% of the enterprises are owned by foreign nationals. As mentioned, these data are not representative of the countries, but reflect that employer characteristics can vary regionally.

**Table 1:** Employer characteristics.

	Kenya	Namibia	Tanzania	Zambia
***Type of employer***				
Enterprise	0.74	0.62	0.95	0.75
Public institution	0.23	0.35	0.05	0.19
Non-governmental organization (NGO)	0.04	0.03	0.01	0.05
***Formal registration***				
Organization is formally registered	0.79	0.66	0.59	0.82
***Start of business operations (if enterprise)***				
Less than 1 year ago	0.01	0.02	0.15	0.07
1-5 years ago	0.41	0.14	0.42	0.26
5-10 years ago	0.23	0.26	0.25	0.27
10-20 years ago	0.23	0.26	0.13	0.28
More than 20 years ago	0.12	0.31	0.04	0.13
***Ownership (if enterprise)***				
Local to the area	0.97	0.85	0.80	0.70
Foreign to the area, but national of a country	0.02	0.10	0.19	0.01
Foreign to country, from a different country	0.01	0.05	0.00	0.22
Joint, a country national and a foreigner	0.00	0.00	0.00	0.07
***Trade association (if enterprise)***				
Belongs to a trade association or employers’ group	0.10	0.13	0.06	0.21
Belong to a trade union	0.04	0.09	0.03	0.15
Practices collective bargaining with the union	0.75	0.88	0.57	0.62
Observations	136	140	220	114

Note: Data from employer survey. The study regions are not nationally representative; thus, direct comparisons between countries should not be made. The figures serve as illustrative contrasts to emphasize contextual differences among the study sites.

Affiliation with trade associations, employer groups, or trade unions is relatively low across all four countries, which is unsurprising given the small-scale nature of most rural enterprises. However, among those businesses that are union-affiliated, collective bargaining is widely practiced, indicating that trade unions, where present, play an important role in influencing labor relations. It is also worth mentioning that a large proportion of the enterprises is still young. Around 33% started their business in the last 5 years, and more than 50% in the last 10 years.

### 3.2. Individuals in employment

Table 2 presents the proportions of individuals participating in various employment activities and their socioeconomic characteristics. On-farm self-employment remains the dominant activity in the rural study areas, accounting for 54% of the adult individuals surveyed across the four countries. Around 10% of the adult individuals are self-employed in non-agricultural businesses, and only 7% are wage-employed. It is important to note that these figures are based on individual-level data. Household-level participation in wage employment is somewhat higher, because most households include more than one adult member.

**Table 2:** Proportion of adult individuals involved in different employment activities.

	Pooled	Kenya	Namibia	Tanzania	Zambia
***Panel A: Employment participation***					
On-farm self-employment	0.54	0.49	0.17	0.74	0.75
Off-farm self-employment	0.10	0.10	0.06	0.16	0.07
Wage employment	0.07	0.11	0.06	0.05	0.04
Participation in any employment	0.61	0.61	0.27	0.77	0.77
Observations	6,722	1,717	1,765	2,086	1,154
***Panel B: Characteristics of wage-employed individuals***					
Sex (1 = male)	0.65	0.63	0.67	0.66	0.67
Age (years)	38.61	37.59	40.11	37.45	40.31
Level of education (years)	10.68	10.61	11.25	9.34	11.53
Experience (years in employment)	8.52	9.43	7.22	8.74	7.88
In-job training (1 = yes)	0.37	0.42	0.42	0.19	0.41
Employment location (share):					
Rural	0.77	0.69	0.79	0.91	0.80
Town within the district	0.10	0.12	0.14	0.05	0.06
Town outside the district	0.12	0.18	0.07	0.04	0.14
Observations	444	185	110	100	49
***Panel C: Wage employed individuals by sectors***					
Agriculture	0.23	0.23	0.10	0.53	0.10
Industry	0.08	0.08	0.06	0.08	0.10
Construction	0.05	0.03	0.05	0.07	0.06
Commerce	0.05	0.06	0.08	0.01	0.04
Hospitality	0.09	0.04	0.26	0.04	0.04
Transport	0.04	0.05	0.00	0.07	0.02
Public administration	0.05	0.06	0.06	0.02	0.04
Education	0.19	0.22	0.14	0.07	0.31
Health	0.04	0.03	0.05	0.04	0.04
Other services	0.21	0.24	0.22	0.09	0.24
Observations	444	185	110	100	49

Note: Data from household survey. Individuals 18 years and older are included. Panel A includes all adult individuals surveyed. Panels B and C only include individuals engaged in wage employment. The proportions shown in the “pooled” column represent the weighted averages across the four countries, with each country having a weight of 0.25. The study regions are not nationally representative; thus, direct comparisons between countries should not be made. The figures serve as illustrative contrasts to emphasize contextual differences among the study sites.

Also important to note is that a sizeable proportion of individuals is not involved in any employment activity because our sample also includes older adults who receive pensions and transfers or depend on the income earned by younger household members. Furthermore, remittances are an important component of rural incomes in all four study countries [6]. In the study region in Namibia, only 27% of the adults surveyed were engaged in any employment at the time of the survey in 2023. This low rate also reflects prolonged drought conditions in the Zambezi region, which has severely impacted agriculture, the region’s primary economic activity [38].

Panel B of Table 2 shows individual characteristics of those who are wage employed. Across all four study countries, men account for about two-thirds of all wage-employed individuals, pointing at gender disparities in rural labor markets. With an average of 11 years of education, most wage-employed individuals have completed secondary school, yet without pursuing higher education.

Panel C of Table 2 shows the distribution of wage-employed individuals across various sectors. Across the four study areas, agriculture is the most important sector, providing jobs for around one-quarter of those who are wage employed, yet with notable differences between the regions. In the Tanzanian study regions, for example, about 53% of all wage employment is in agriculture, whereas in the Namibian and Zambian study regions, this share is only 10%. Education and industry are relevant sectors for wage employment in all study areas. The hospitality sector is especially important in the Namibian study region, reflecting its proximity to a national park with various tourism activities. Wage employment in “other services” is relevant in all study regions and includes a wide variety of activities in entertainment, recreation, cleaning, and personal services such as hairdressers and beauty salons.

### 3.3. Wages

**Fig 1:**
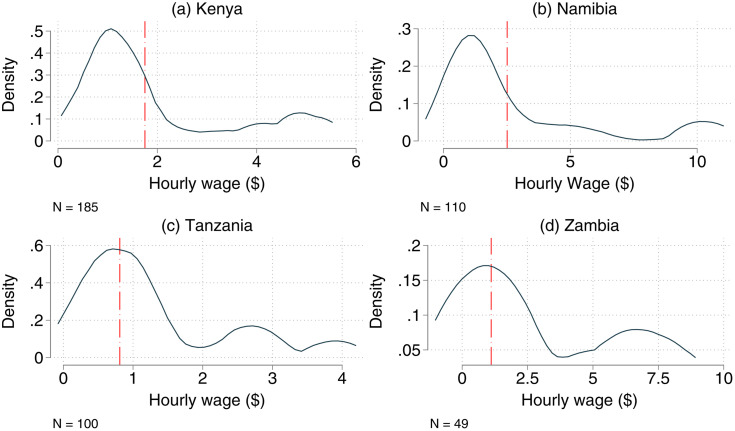
Individual wages received (per hour in PPP dollars). The kernel density estimates show the distribution of hourly wages of individuals engaged in wage employment based on household survey data. N denotes the number of observations for each country. The red vertical dotted lines indicate the national minimum wage for each country. The study regions are not nationally representative; thus, direct comparisons between countries should not be made. The figures serve as illustrative contrasts to emphasize contextual differences among the study sites.

Fig 1 presents density plots of the hourly wages received by individuals engaged in wage employment, differentiated by country/region (further details about the average number of hours worked and wages received are shown in Tables D-H in S1 File). For each country, the national minimum wage is also shown. In the study region in Kenya (panel a), a peak of the distribution function is observed at $1.16, which is well below the minimum wage of $1.75. In fact, only 31% of the wage-employed individuals in Kenya achieve the national minimum wage. The situation is similar in the study region in Namibia (panel b), where only 30% of all wage-employed individuals achieve the national minimum wage of $2.51.

In the study region in Tanzania (panel c of Fig 1), the peak of the wage distribution is closer to the national minimum wage of $0.81, which is achieved by 59% of the wage-employed individuals in our sample. In Zambia (panel d), 47% of the workers achieve the minimum wage of $1.11. In all study regions across the four countries, we observe smaller peaks in the right tails of the distributions, indicating that some individuals are also involved in higher-paying employment.

**Fig 2:**
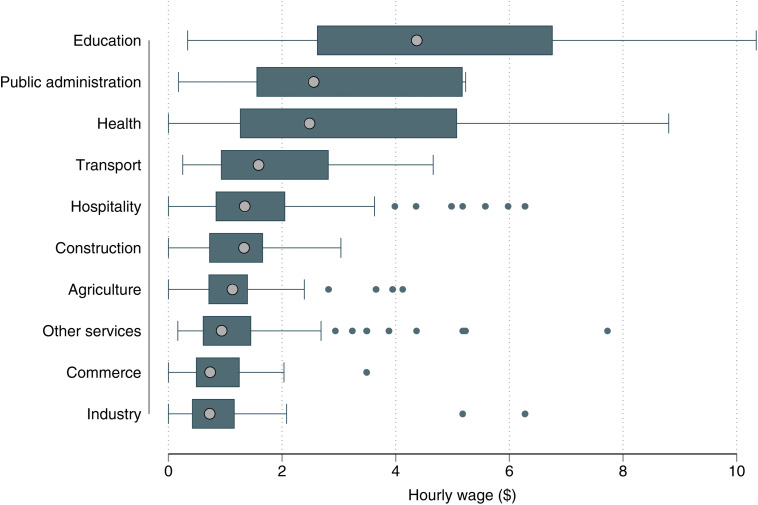
Individual wages received by sector (per hour in PPP dollars). Pooled data from the household survey across the four study countries with 444 individuals engaged in wage employment. The box plots show the wage distributions. The grey dot within each box represents the median wage, while the left and right boundaries of the box indicate the 25^th^ and 75^th^ percentiles, respectively. The ‘whiskers’ show the range of wages beyond the middle 50% of workers.

Fig 2 pools the data across the regions in the four countries but disaggregates by sector (for a disaggregation by country and sector, see Tables G and H in S1 File). We find that workers in education, public administration, and health tend to earn higher wages than those in other sectors. This can be attributed to the more specialized qualifications typically required, even though considerable variation also exists within each sector, likely due to differing responsibilities and levels of experience.

The lowest average wages are earned by workers in industry and commerce (Fig 2), where most of the jobs do not require specific skills. Workers in agriculture and “other services” also receive quite low wages on average. Important to note is that the daily wages in agriculture are often lower than in commerce or industry, but because of fewer working hours per day (see Table E in S1 File), the mean hourly wages in agriculture are somewhat higher. Work in agriculture is typically also more seasonal than in other sectors, which needs to be considered when extrapolating the hourly wages to monthly or annual incomes.

### 3.4. Employment contracts

**Fig 3:**
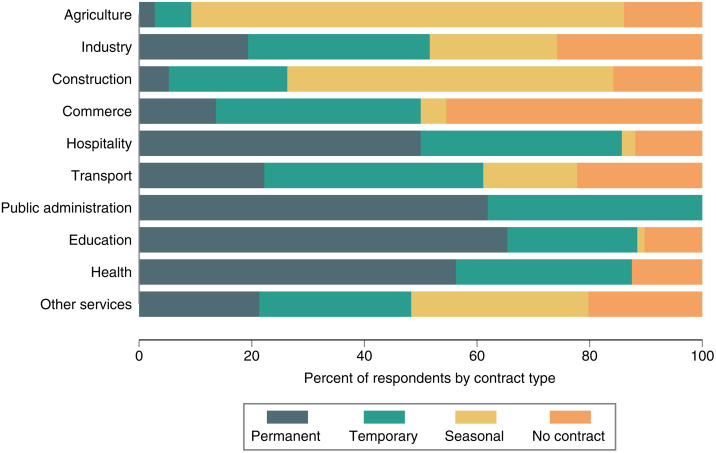
Prevalence of different types of employment contracts by sector. Pooled data from the household survey across the four study countries with 444 individuals engaged in wage employment.

Beyond wages, an additional key aspect of labor conditions is employment stability. We, therefore, assess what type of employment contracts and agreements workers in rural Africa have. Fig 3 illustrates the prevalence of different contract types by sector, based on the household survey data. The results show high variation of different contract types both within and across sectors. In the commerce sector, for example, nearly half of the workers operate without any formal contract. This is due to the large prevalence of informal jobs in rural areas, where small businesses, family-owned shops, and street vending are common. In these settings, employment is often based on casual work relationships and verbal agreements. Also in most sectors other than commerce, employment relationships without formal contracts are common.

In the agricultural sector, workers often engage in seasonal employment, typically being hired for specific tasks like land preparation, planting, or harvesting, depending on the agricultural cycle. While both agriculture and commerce share the characteristics of informal employment, the key difference lies in the seasonality of agricultural jobs, as opposed to the more continuous, yet equally unstructured, nature of work in the commerce sector.

Permanent contracts are more common in public administration, and the health and education sectors, where public organizations typically dominate. These sectors offer more stable job arrangements with formal contracts, providing greater job security, social benefits, and legal protection than commerce and agriculture.

### 3.5. Decent work

**Fig 4:**
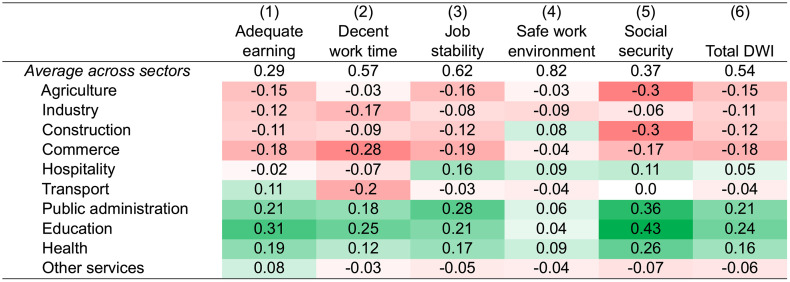
Decent work indicators by sector. The decent work index (DWI) and each of its indicators can range from 0 to 1 (first row). All other cells represent the deviation from the mean of the indicator across sectors. Significant negative deviations are shaded in red (worse than average conditions), significant positive ones in green (better than average conditions), whereby increasing color intensities indicate larger deviations. Pooled data from the household survey across the four study countries with 444 individuals engaged in wage employment.

Job conditions can also be influenced by various other aspects, such as work time, occupational safety, or social benefits. As explained above, we use various indicators to construct a DWI. Fig 4 shows the mean DWI in column (6) and the five indicators in columns (1) to (5). The overall mean DWI of 0.54 reveals that the typical labor conditions in rural Africa are fairly poor, with many of the conditions for decent work not being met. The “adequate earning” (column 1) and “social security” (column 5) indicators show particularly low values on average (first row).

Fig 4 also shows some interesting differences in labor conditions between sectors. Education and public administration consistently rank highest across all decent work indicators. Most schools in rural Africa are government-run, ensuring that workers (e.g., teachers, admin and technical staff) benefit from formal employment procedures, which provide at least minimum wages, job stability, decent work times, and certain social benefits. Similarly, the health sector, also largely managed by the government, performs relatively well in terms of these indicators. In contrast, commerce and agriculture have the lowest decent work scores, followed by industry and construction.

Interestingly, occupational safety (column 4 in Fig 4) is relatively high across most sectors, including agriculture and construction. Highly toxic chemicals are not widely used in the mainly subsistence-oriented local agricultural systems, and most of the rural construction activities are smaller-scale projects with lower risks than larger urban construction sites.

### 3.6. Job satisfaction

**Fig 5:**
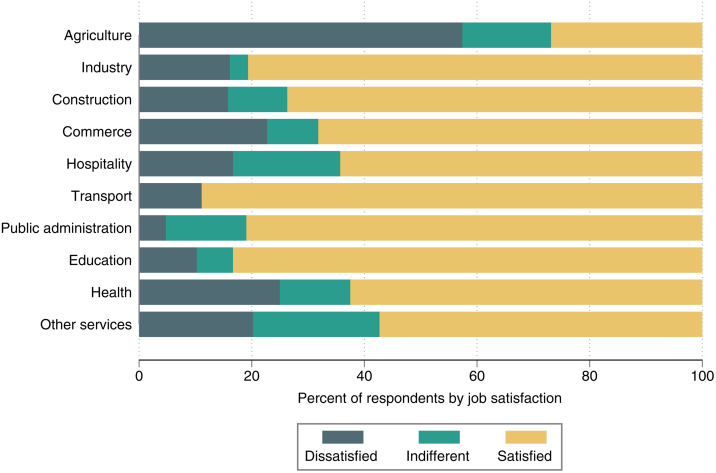
Individual job satisfaction by sector. Pooled data from the household survey across the four study countries with 444 individuals engaged in wage employment.

In spite of payment below the national minimum wage for the majority of rural workers and poor labor conditions also in terms of other objectively measurable indicators, individual job satisfaction is relatively high. Fig 5 reveals that in most sectors more than 60% of the individuals in wage employment are satisfied with their current work. The only exception is the agricultural sector, where close to 60% of the wage-employed individuals are dissatisfied, and another 15% are indifferent.

These patterns offer interesting insights. Many people in rural Africa do not have access to jobs beyond self-employed activities in farming and small own businesses. Agricultural employment opportunities on other farms may sometimes be available. But agricultural employment depends on weather conditions, is seasonal at best, and therefore not a very satisfying situation. Hence, the mere fact of having wage employment in other sectors can be a source of satisfaction, even when the working conditions are far from perfect.

Fig 5 further shows that in the health, commerce, and “other services” sectors, dissatisfaction rates are also around 20% or slightly above. This is low in comparison to agriculture, but still noteworthy, especially with respect to the health sector. As shown above, wages and other work conditions in the health sector are consistently better than in other rural sectors. Evidently, objective indicators of labor conditions are not the only factors that matter for individual job satisfaction. Hence, it is useful to look at job satisfaction in addition to the DWI.

### 3.7. Determinants of wages and decent work

We now use the regression models explained above to analyze determinants of labor conditions. Table 3 shows regression results from models using the individual-level data, with wages and the DWI as outcome variables. As can be seen, individual education and experience of the worker significantly increase hourly wages, highlighting the importance of skills in boosting earning potentials. Job training also plays a key role in enhancing both wages and the DWI. Trained workers tend to be more efficient and capable of handling complex tasks, leading to better job conditions. Age is also positively associated with the DWI.

**Table 3:** Factors influencing individual labor conditions.

	(1)	(2)
	Hourly wage (PPP $)	Decent work index (DWI)
Sex (1 = male)	0.098	−0.009
	(0.172)	(0.016)
Age (years)	−0.008	0.002***
	(0.007)	(0.001)
Level of education (years)	0.058**	0.004
	(0.024)	(0.003)
Experience (years)	0.027**	0.001
	(0.012)	(0.001)
In-job training (1 = yes)	0.840***	0.164***
	(0.226)	(0.021)
*Employment location in rural as a reference*		
Town within the district	0.286	0.051**
	(0.273)	(0.026)
Town outside the district	0.364*	0.058**
	(0.206)	(0.028)
*Sector dummies (agriculture is the reference)*		
Industry	−0.887***	−0.015
	(0.231)	(0.037)
Construction	−0.541*	−0.020
	(0.281)	(0.025)
Commerce	−0.893***	−0.067
	(0.304)	(0.042)
Hospitality	−0.402	0.076**
	(0.301)	(0.031)
Transport	0.045	−0.002
	(0.308)	(0.053)
Public administration	0.599	0.211***
	(0.401)	(0.035)
Education	2.363***	0.219***
	(0.393)	(0.033)
Health	0.698	0.147***
	(0.581)	(0.049)
Other services	−0.238	0.041*
	(0.178)	(0.021)
Constant	0.484	0.240***
	(0.388)	(0.041)
Country fixed effects	Yes	Yes
R-squared	0.499	0.590
Observations	444	444

Note: Coefficient estimates from OLS regressions are shown with robust standard errors in parentheses. Pooled data from the household survey across the four study countries, only including individuals engaged in wage employment. * Significant at the 10% level; ** Significant at the 5% level; *** Significant at the 1% level.

Additionally, workers living in rural areas but employed in towns or cities outside of their district earn more and have better working conditions than those working within their own rural districts. This likely stems from better infrastructure and broader job opportunities available in urban areas, associated with higher wages and better labor conditions.

In terms of sectoral differences, Table 3 shows that workers in industry, construction, and commerce earn lower hourly wages than those in agriculture, while workers in the education sector earn by far the highest wages. These findings align with the results observed in Fig 2. Furthermore, workers in public administration, education, and health have a higher DWI than those in agriculture, largely due to more stable contracts and social benefits such as paid leave and health insurance coverage.

Table 4 shows additional factors that influence wages from a regression model with the employer data. As can be seen, public institutions and NGOs pay significantly higher hourly wages than private enterprises. Registered and unionized employers also pay higher average wages, as do enterprises with foreign or joint domestic-foreign ownership. Larger employers and those with longer operational histories also provide higher wages. These findings highlight the strong impact of employer characteristics on labor conditions.

**Table 4:** Employer characteristics influencing average wage rates.

	Hourly wage (log)
Public institution	0.522***
	(0.145)
Non-governmental organization (NGO)	0.754***
	(0.267)
Number of employees	0.003**
	(0.001)
Foreign or jointly-owned enterprise	0.314**
	(0.135)
Business registration	0.243***
	(0.092)
Trade union	0.567***
	(0.107)
***Business operation***	
1–5 years	0.349**
	(0.160)
5–10 years	0.412**
	(0.169)
10–20 years	0.430**
	(0.171)
More than 20 years	0.622***
	(0.180)
Constant	−0.306
	(0.197)
Sector dummies	Yes
Country fixed effects	Yes
R-squared	0.417
Observations	610

Note: OLS coefficient estimates are shown with robust standard errors in parentheses. Data from employer survey pooled across the four countries. Wages represent the average wage paid by each employer. ** Significant at the 5% level; *** Significant at the 1% level.

### 3.8. Determinants of job satisfaction

We now analyze factors influencing individual job satisfaction, using an ordered logit model. Table 5 shows that, individuals who received in-job training are more likely to be satisfied with their jobs, as training enhances both their skills and performance, leading to increased job fulfillment, advancement opportunities, and higher wages, as highlighted above. Additionally, workers employed in towns, whether within or outside the own district, are more likely to be satisfied with their jobs than those working in rural areas, probably because urban areas have more attractive job opportunities.

**Table 5:** Ordered logit regression for job satisfaction (marginal effects).

	(1)	(2)	(3)
	Dissatisfied	Indifferent	Satisfied
Sex (1 = male)	−0.046	−0.009	0.055
	(0.037)	(0.007)	(0.044)
Age (years)	0.000	0.000	−0.000
	(0.001)	(0.000)	(0.002)
Level of education (years)	0.010*	0.002*	−0.012*
	(0.005)	(0.001)	(0.006)
Decent work index (DWI)	−0.262**	−0.049**	0.311**
	(0.123)	(0.023)	(0.144)
Experience (years)	−0.003	−0.001	0.003
	(0.002)	(0.000)	(0.003)
In-job training (1 = yes)	−0.142***	−0.027***	0.169***
	(0.050)	(0.009)	(0.058)
*Employment location in rural as the base*			
Town within the district	−0.129*	−0.024**	0.154**
	(0.067)	(0.012)	(0.078)
Town outside the district	−0.164**	−0.031**	0.194**
	(0.064)	(0.013)	(0.076)
*Sector dummies (agriculture as the reference)*			
Industry	−0.311***	−0.073**	0.384***
	(0.067)	(0.033)	(0.092)
Construction	−0.290***	−0.061*	0.351***
	(0.078)	(0.035)	(0.107)
Commerce	−0.261***	−0.046	0.307***
	(0.086)	(0.034)	(0.116)
Hospitality	−0.181**	−0.018	0.198**
	(0.080)	(0.014)	(0.091)
Transport	−0.351***	−0.102**	0.453***
	(0.069)	(0.047)	(0.106)
Public administration	−0.236**	−0.035	0.271*
	(0.112)	(0.038)	(0.146)
Education	−0.261***	−0.046	0.307***
	(0.087)	(0.030)	(0.113)
Health	−0.100	−0.004	0.104
	(0.154)	(0.015)	(0.168)
Other services	−0.186***	−0.019*	0.205***
	(0.060)	(0.010)	(0.066)
Country fixed effects	Yes	Yes	Yes
Observations	444	444	444

Note: Average marginal effects are shown with standard errors in parentheses. Pooled data from the household survey across the four study countries, only including individuals engaged in wage employment. * Significant at the 10% level; ** Significant at the 5% level; *** Significant at the 1% level.

Another key finding from the estimates in Table 5 is that higher scores on the DWI are associated with higher levels of job satisfaction. This suggests that better working conditions, such as job security, social protection, and fair wages, play an important role for job satisfaction, as one would expect. Strikingly, higher levels of education are associated with a greater likelihood of job dissatisfaction or indifference. This could be because highly educated individuals often have higher expectations for job roles, wages, and career progression, which, when unmet, can lead to dissatisfaction. Additionally, while better-educated individuals typically enjoy greater job resources such as higher wages and job autonomy, they may also face greater job demands, including longer work hours, task pressure, and job intensity. As Solomon et al. [39] note, such job demands can offset the positive gains from greater resources, leading to higher stress and lower satisfaction.

The lower part of Table 5 shows that individuals working in industry, construction, commerce, hospitality, transport, public administration, education, and other services are more likely to be satisfied with their jobs than those with wage employment in agriculture. This is consistent with the findings in Fig 5. Interesting to see is that these results also hold after controlling for DWI and other individual characteristics.

## 4. Conclusion

Wage employment in rural Africa has, so far, received relatively little attention, despite its rising importance as a source of livelihoods beyond subsistence-oriented agriculture. Historically, most rural households in sub-Saharan Africa relied on subsistence farming, sometimes supplemented by self-employed activities including petty trading, artisanal crafts, or food vending, among others. However, more recently, with further population growth, land and technology limitations, and climate change, the demand for other types of employment in rural Africa has been rising, to contribute to income generation and stability. New wage employment opportunities have also emerged in a variety of sectors, even though these opportunities and the associated labor demand often fall short of the rising rural labor supply [1–6].

Our study, utilizing data from actual and potential workers and employers in rural regions of Kenya, Namibia, Tanzania, and Zambia, evaluates the prevalence of wage employment and examines the labor conditions across relevant sectors. In particular, we have analyzed employment wages, contract types, working hours, occupational safety, and other social benefits for the calculation of a decent work index. We have also analyzed individual job satisfaction, as well as factors influencing the labor conditions.

Based on our data, we find that only 7% of the adult population in rural areas are wage employed, whereas close to 60% are self-employed in agriculture or small non-agricultural businesses. For those in wage employment, more than 60% earn less than the national minimum wage. The mean decent work index of 0.54 suggests that the average labor conditions are fairly poor.

While agriculture, hospitality, and other services are locally the most relevant sectors for wage employment, jobs in these sectors are typically associated with low wages and poor working conditions. In contrast, public administration, education, and the healthcare sector offer higher levels of job security, better wages, and other social benefits. Most workers in these sectors are government employees with permanent contracts, health insurance coverage, paid leave, and access to formal pension schemes.

Our regression analysis shows that higher levels of education, in-job training, and job experience are positively associated with wages and decent work conditions. However, we also find that higher educational qualifications do not necessarily result in higher job satisfaction. On the contrary, our results suggest that – after controlling for other work conditions – higher education is associated with more job dissatisfaction. This pattern may be explained by the fact that better-educated people often have higher job expectations, which cannot always be met by the available job opportunities in rural Africa.

We have also identified employer-related factors influencing labor conditions. Specifically, we find that public institutions and NGOs pay higher wages than private enterprises, likely because many of the jobs in small and medium enterprises in rural Africa are informal. Larger enterprises, especially those involving foreign ownership structures, pay higher wages, as do formally registered and unionized employers. The age of an operation is also positively associated with average wage levels.

We recognize that our study areas are not representative of Kenya, Namibia, Tanzania, and Zambia, as we only surveyed specific regions within these countries. The regions surveyed are among the poorer ones within each country, which may limit the external validity of our findings in several ways. First, the proportion of individuals engaged in wage employment is likely higher in more economically developed areas with better market access and infrastructure, so our 7% wage employment rate might underestimate national averages. Second, wage levels, job security, and social benefits may be higher in peri-urban and vibrant rural areas with more businesses and labor competition. Third, the presence of formal employment contracts, workplace safety measures, and social benefits—key components of our decent work index—is likely more prevalent in regions with stronger labor inspection agencies and proximity to urban administrative centers. As a result, our findings are most relevant to remote, economically marginalized rural areas of sub-Saharan Africa. The lack of comparable nationally representative data makes it hard to measure these differences, highlighting the need for broader labor market surveys in rural Africa. Still, our results offer valuable insights into labor conditions in disadvantaged rural areas where employment issues are likely most severe.

However, we feel that we cover a variety of conditions that are typical for rural areas of sub-Saharan Africa, so the general findings offer some broader lessons for research and policy. Formal employment opportunities – especially in sectors other than agriculture – are insufficiently available in many parts of rural Africa. Moreover, most of the available jobs have relatively poor labor conditions. There is an urgent need to generate more jobs, especially decent ones, through appropriate rural development policies.

Based on our findings, we suggest several targeted strategies. First, since larger and registered employers tend to offer significantly higher wages, policies should aim to formalize businesses and promote growth via simpler registration procedures and focused support for expanding small enterprises. Second, given that education, training, and experience are positively linked to higher wages and better working conditions, expanding access to skills development—particularly vocational training aligned with local labor market needs—can directly increase worker productivity and income.

Third, our results show a notable gap in terms of wages and working condition between public institutions and private firms, with many private enterprises operating informally. Strengthening labor standards enforcement and encouraging the private sector to adhere to minimum wage laws and basic worker protection could significantly improve the working conditions for rural workers in private enterprises. Lastly, investing in infrastructure—such as roads, energy, water, and communication networks—is essential to reduce operational costs and attract more enterprises to rural areas. Implementing these strategies could help reduce poverty, increase resilience, and curb the massive rural-urban migration trend to some extent.

## Supporting information

S1 File Supporting information. (DOCX)

## References

[1] Christiaensen L, Maertens M. Rural employment in Africa: trends and challenges. Annu Rev Resour Econ. 2022;14(1):267–89. doi: 10.1146/annurev-resource-111820-014312

[2] Davis B, Di Giuseppe S, Zezza A. Are African households (not) leaving agriculture? Patterns of households’ income sources in rural Sub-Saharan Africa. Food Policy. 2017;67:153–74. doi: 10.1016/j.foodpol.2016.09.018 28413253 PMC5384437

[3] Gindling TH, Newhouse D. Self-employment in the developing world. World Development. 2014;56:313–31. doi: 10.1016/j.worlddev.2013.03.003

[4] Khan R, Morrissey O. Income diversification and household welfare in Uganda 1992–2012. Food Policy. 2023;116:102421. doi: 10.1016/j.foodpol.2023.102421

[5] Van den Broeck G, Kilic T. Dynamics of off-farm employment in sub-saharan africa: a gender perspective. World Development. 2019;119:81–99. doi: 10.1016/j.worlddev.2019.03.008

[6] Mutsami C, Parlasca MC, Qaim M. Evolving farm and off‐farm income sources and jobs in rural Africa. J of Intl Development. 2025;37(6):1367–80. doi: 10.1002/jid.70010

[7] Timmer CP. A world without agriculture: The structural transformation in historical perspective. Washington, DC: Aei Press. 2009.

[8] Losch B, Freguin-Gresh S, White ET. Structural transformation and rural change revisited: challenges for late developing countries in a globalizing world. World Bank. 2012. https://agritrop.cirad.fr/566345/

[9] McMillan M, Rodrik D, Verduzco-Gallo Í. Globalization, structural change, and productivity growth, with an update on Africa. World Development. 2014;63:11–32. doi: 10.1016/j.worlddev.2013.10.012

[10] Barrett CB, Christiaensen L, Sheahan M, Shimeles A. On the structural transformation of rural Africa. Journal of African Economies. 2017;26(suppl_1):i11–35. doi: 10.1093/jae/ejx009

[11] Müller C, Cramer W, Hare WL, Lotze-Campen H. Climate change risks for African agriculture. Proc Natl Acad Sci U S A. 2011;108(11):4313–5. doi: 10.1073/pnas.1015078108 21368199 PMC3060257

[12] Muyanga M, Jayne TS. Effects of rising rural population density on smallholder agriculture in Kenya. Food Policy. 2014;48:98–113. doi: 10.1016/j.foodpol.2014.03.001

[13] Musungu AL, Kubik Z, Qaim M. Drought shocks and labour reallocation in rural Africa: evidence from Ethiopia. European Review of Agricultural Economics. 2024;51(4):1045–68. doi: 10.1093/erae/jbae020

[14] Apraku A, Morton JF, Apraku Gyampoh B. Climate change and small-scale agriculture in Africa: does indigenous knowledge matter? Insights from Kenya and South Africa. Scientific African. 2021;12:e00821. doi: 10.1016/j.sciaf.2021.e00821

[15] Dedehouanou SFA, Araar A, Ousseini A, Harouna AL, Jabir M. Spillovers from off-farm self-employment opportunities in rural Niger. World Development. 2018;105:428–42. doi: 10.1016/j.worlddev.2017.12.005

[16] Drall A, Mandal SK. Investigating the existence of entry barriers in rural non-farm sector (RNFS) employment in India: a theoretical modelling and an empirical analysis. World Development. 2021;141:105381. doi: 10.1016/j.worlddev.2020.105381

[17] Sen B, Dorosh P, Ahmed M. Moving out of agriculture in Bangladesh: The role of farm, non-farm and mixed households. World Development. 2021;144:105479. doi: 10.1016/j.worlddev.2021.105479

[18] Van den Broeck G, Van Hoyweghen K, Maertens M. Employment conditions in the senegalese horticultural export industry: a worker perspective. Development Policy Review. 2016;34(2):301–19. doi: 10.1111/dpr.12153

[19] Ehlert CR, Mithöfer D, Waibel H. Worker welfare on Kenyan export vegetable farms. Food Policy. 2014;46:66–73. doi: 10.1016/j.foodpol.2014.01.004

[20] Krumbiegel K, Maertens M, Wollni M. The role of fairtrade certification for wages and job satisfaction of plantation workers. World Development. 2018;102:195–212. doi: 10.1016/j.worlddev.2017.09.020

[21] Staelens L, Desiere S, Louche C, D’Haese M. Predicting job satisfaction and workers’ intentions to leave at the bottom of the high value agricultural chain: evidence from the Ethiopian cut flower industry. Int J Human Resource Manag. 2016;29(9):1609–35. doi: 10.1080/09585192.2016.1253032

[22] Suzuki A, Mano Y, Abebe G. Earnings, savings, and job satisfaction in a labor-intensive export sector: Evidence from the cut flower industry in Ethiopia. World Development. 2018;110:176–91. doi: 10.1016/j.worlddev.2018.05.029

[23] Meemken E-M, Sellare J, Kouame CN, Qaim M. Effects of Fairtrade on the livelihoods of poor rural workers. Nat Sustain. 2019;2(7):635–42. doi: 10.1038/s41893-019-0311-5

[24] Fabry A, Van den Broeck G, Maertens M. Decent work in global food value chains: Evidence from Senegal. World Development. 2022;152:105790. doi: 10.1016/j.worlddev.2021.105790

[25] KNBS. Kenya population and housing census. 2019. https://www.knbs.or.ke/2019-kenya-population-and-housing-census-results/

[26] Jha S, Kaechele H, Sieber S. Factors influencing the adoption of agroforestry by smallholder farmer households in Tanzania: Case studies from Morogoro and Dodoma. Land Use Policy. 2021;103:105308. doi: 10.1016/j.landusepol.2021.105308

[27] ZamStats. 2022 Census of Population and Housing. Zambia Statistics Agency. 2022. https://www.zamstats.gov.zm/wp-content/uploads/2023/12/2022-Census-of-Population-and-Housing-Preliminary.pdf

[28] Bonnet F, Figueiredo JB, Standing G. A family of decent work indexes. Int Labour Rev. 2003;142(2):213–38. doi: 10.1111/j.1564-913x.2003.tb00259.x

[29] Schuster M, Vranken L, Maertens M. You Can(’t) always get the job you want: employment preferences in the peruvian horticultural export Chain. J Development Stud. 2019;56(7):1408–29. doi: 10.1080/00220388.2019.1666976

[30] Decent work indicators: Guidelines for producers and users of statistical and legal framework indicators. Geneva, Switzerland: International Labor Office. 2013. https://www.ilo.org/publications/decent-work-indicators-guidelines-producers-and-users-statistical-and-legal

[31] Burchell B, Sehnbruch K, Piasna A, Agloni N. The quality of employment and decent work: definitions, methodologies, and ongoing debates. Cambridge J Econ. 2013;38(2):459–77. doi: 10.1093/cje/bet067

[32] Pyatt G, Becker GS. Human capital: a theoretical and empirical analysis, with special reference to education. The Economic J. 1966;76(303):635. doi: 10.2307/2229541

[33] Mincer JA. Schooling, experience, and earnings. NBER. 1974. https://www.nber.org/books-and-chapters/schooling-experience-and-earnings

[34] Shapiro C, Stiglitz JE. Equilibrium unemployment as a worker discipline device. Am Econ Rev. 1984;74:433–44. Available: https://www.jstor.org/stable/1804018

[35] Rosen S. Chapter 12 The theory of equalizing differences. Handbook of Labor Economics. Elsevier. 1986. p. 641–92. doi: 10.1016/s1573-4463(86)01015-5

[36] Koenker R, Hallock KF. Quantile regression. J Economic Perspectives. 2001;15(4):143–56. doi: 10.1257/jep.15.4.143

[37] Alkire S, Santos ME. Acute multidimensional poverty: A new index for developing countries. 38. Oxford Poverty & Human Development Initiative (OPHI), Oxford Department of International Development, Queen Elizabeth House (QEH), University of Oxford. 2010. https://ophi.org.uk/sites/default/files/2024-03/OPHI-wp38_with_note.pdf

[38] National Planning Commission. The root causes of unemployment and possible policy interventions. Republic of Namibia. 2020. https://www.npc.gov.na/wp-content/uploads/2023/06/The-Root-Causes-of-Unemployment-and-Posssible-Policy-Interventions-2020.pdf

[39] Solomon BC, Nikolaev BN, Shepherd DA. Does educational attainment promote job satisfaction? The bittersweet trade-offs between job resources, demands, and stress. J Appl Psychol. 2022;107(7):1227–41. doi: 10.1037/apl0000904 35737558

