# Supplementary material for: An inquiry into labor conditions across key rural sectors in Africa
Source: PLoS One. 2025 Dec 22;20(12):e0338694. doi: 10.1371/journal.pone.0338694 (PMC12721550; doi:10.1371/journal.pone.0338694)
Supplement: S1 File — (DOCX) [file pone.0338694.s001.docx]

**Supporting information**

**1. Decent work index calculation and validation**

**Table A. Decent work index calculation**

| ***Indicator*** | | ***Definition*** | ***Calculation*** |
| --- | --- | --- | --- |
| ***1) Adequate earnings and productive wage*** | | | |
|  | Minimum wage | The worker earns at least the minimum wage | 1 if yes  0 if otherwise |
|  | Fringe benefits | The worker receives fringe benefits (housing, meals or transport) | 1 if 3 benefits  0.7 if 2 benefits  0.3 if 1 benefit  0 if otherwise |
|  | Training | Worker receives job training | 1 if yes  0 if otherwise |
| ***2) Decent work time*** | | | |
|  | Weekly hours | The worker works 48 hours or less per week | 1 if yes  0 if otherwise |
|  | Paid leave | The worker receives a paid annual leave | 1 if yes  0 if otherwise |
|  | Unsocial hours | A worker who undertakes night shifts or works on public holidays is appropriately compensated for the extra time | 1 if yes  0 if otherwise |
| ***3) Stability and security of work*** | | | |
|  | Contract type | Type of contract the worker has | 1 if permanent  0.75 if temporary  0.5 if seasonal  0 if no contract |
| ***4) Safe work environment*** | | | |
|  | Work accidents | The worker has had no work accidents since joining the current employer | 1 if yes  0 if otherwise |
|  | Dangerous products | The worker's occupation does not involve handling dangerous products (e.g. pesticides, other toxic materials) without protection | 1 if yes  0 if otherwise |
| ***5) Social security*** | | | |
|  | Insurance | The worker receives medical insurance | 1 if yes  0 otherwise |
|  | Sick leave | The worker can get paid sick leave whenever necessary | 1 if yes  0 otherwise |
|  | Paternity/maternity leave | The worker can get paternity/maternity leave | 1 if yes  0 otherwise |

Note: The index is based on the guidelines put forward by the International Labor Organization (ILO, 2013); however, due to the unavailability of some indicators, modifications were made to adapt the index to the available data.

**1.1 Decent work index validation**

To ensure the robustness of our decent work index, we conducted two complementary validation analyses: internal consistency to assess whether the five components are related in a meaningful way and can be combined into a single index using Cronbach's alpha, and dimensionality assessment with principal component analysis (PCA) to determine whether the five components share a common underlying dimension of job quality or are unrelated aspects.

***Internal consistency***

We calculate Cronbach’s Alpha to evaluate the reliability of our five-component Decent Work Index, which includes adequate earnings, decent working time, job stability, a safe work environment, and social security. As shown in Table A2, the index yields a Cronbach’s Alpha of 0.693, based on five items with an average inter-item covariance of 0.034. Interpreting this coefficient does not depend on a strict cutoff. Although a value of 0.70 is often cited as a general guideline for acceptable reliability [1], several studies highlight that the appropriate threshold depends on the construct’s scope, the number of items, and the measurement purpose [2,3].

Tracing back to earlier psychometric research, Nunnally [4] initially suggested alpha levels of 0.50 to 0.60 for early research stages, 0.80 for well-established tools, and 0.90 for clinical use, later adjusting the lower limit to 0.70 in later editions[1,5]. Streiner [6] cautions that very high coefficients—above 0.90—may indicate redundancy among items rather than good internal consistency. Likewise, Tavakol and Dennick [7] observe that alpha values between 0.6 and 0.7 can be acceptable in exploratory, multidimensional scales or those with a small number of theoretically diverse items.

In this context, the Decent Work Index captures several conceptually related yet distinct aspects of employment quality. The coefficient of 0.693, therefore, indicates a reasonable and theoretically justified level of internal consistency: the five dimensions are correlated enough to reflect a coherent latent construct of “decent work,” but not so highly correlated as to suggest redundancy. This balance supports the reliability and conceptual validity of the index, confirming that the selected components serve as complementary dimensions rather than overlapping measures of the same phenomenon.

***Dimensionality Assessment***

To further analyze the structure of the Decent Work Index, we evaluate its dimensionality using Principal Component Analysis (PCA). As shown in Table A2, the first principal component has an eigenvalue of 2.42 and accounts for 48.4% of the total variance across the five indicators. All five components load positively on this first factor, with loadings ranging from 0.10 (safe work environment) to 0.55 (social security). The consistently positive loadings indicate that the indicators reflect a common underlying dimension of job quality, consistent with the broader concept of “decent work.”

**Table B. Decent work index validation metrics**

| **Metric** | **Value** |
| --- | --- |
| ***Internal consistency*** |  |
| Cronbach’s Alpha | 0.693 |
| Number of items | 5 |
| Average inter-item covariance | 0.034 |
| ***Principal component analysis*** |  |
| First component eigenvalue | 2.422 |
| Variance explained (PC1) | 48.4% |
| Variance explained (PC1 + PC2) | 68.8% |
| ***Component loading on PC1*** |  |
| Adequate earnings | 0.531 |
| Decent working time | 0.435 |
| Job stability | 0.467 |
| Safe work environment | 0.100 |
| Social security | 0.549 |

Note: Based on 444 observations from pooled household survey data across the four study countries, including only individuals engaged in wage employment.

The first two principal components together explain 68.8% of the total variance, showing that while the indicators share a common latent dimension, each still provides unique information. The lack of a single dominant loading (the highest being 0.55) supports the idea that the components are complementary but not redundant dimensions of decent work. This reinforces the earlier reliability finding—specifically, that a Cronbach’s Alpha of 0.693 indicates a balanced internal structure: sufficiently correlated to suggest coherence, yet diverse enough to reflect the multifaceted nature of the International Labour Organization’s (ILO) decent work framework. Both the PCA and reliability results strongly justify combining the five items into a composite index of decent work.

**2. Distribution of employers and wages**

**Table C. Sectors of operation of employers (proportions)**

|  | Kenya | Namibia | Tanzania | Zambia |
| --- | --- | --- | --- | --- |
| Agriculture | 0.12 | 0.49 | 0.09 | 0.15 |
| Industry | 0.10 | 0.01 | 0.08 | 0.09 |
| Construction | 0.03 | 0.02 | 0.03 | 0.04 |
| Commerce | 0.28 | 0.18 | 0.33 | 0.27 |
| Hospitality | 0.15 | 0.12 | 0.19 | 0.24 |
| Transport | 0.04 | 0.01 | 0.05 | 0.04 |
| Public administration | 0.03 | 0.17 | 0.02 | 0.03 |
| Education | 0.12 | 0.14 | 0.02 | 0.05 |
| Health | 0.12 | 0.11 | 0.15 | 0.09 |
| Other services | 0.03 | 0.06 | 0.06 | 0.08 |
| Observations | 136 | 140 | 220 | 114 |

Note: Based on employer survey data.

**Table D. Average working hours per day and week**

| Country | Day | | Week | | Observations |
| --- | --- | --- | --- | --- | --- |
|  | Mean | SD* | Mean | SD* |  |
| Kenya | 7.86 | 2.52 | 42.18 | 20.42 | 191 |
| Namibia | 8.99 | 2.82 | 54.69 | 22.63 | 110 |
| Tanzania | 8.43 | 2.55 | 46.42 | 21.77 | 106 |
| Zambia | 8.41 | 2.86 | 44.43 | 14.99 | 49 |

Note: Based on household survey data. Only individuals engaged in wage employment are included.

**Table E. Average working hours per day by sector**

|  | Kenya | | Namibia | | Tanzania | | Zambia | |
| --- | --- | --- | --- | --- | --- | --- | --- | --- |
|  | Mean | SD | Mean | SD | Mean | SD | Mean | SD |
| Agriculture | 6.33 | 1.46 | 8.64 | 2.58 | 7.30 | 2.12 | 6.60 | 2.61 |
| Industry | 7.60 | 3.09 | 10.14 | 3.89 | 8.88 | 2.85 | 9.40 | 2.19 |
| Construction | 7.60 | 0.55 | 9.33 | 1.37 | 9.71 | 2.21 | 8.00 | 0.00 |
| Commerce | 9.73 | 1.90 | 9.78 | 2.17 | 9.00 | . | 7.50 | 0.71 |
| Hospitality | 10.29 | 6.40 | 9.17 | 2.38 | 12.25 | 0.50 | 16.00 | 11.31 |
| Transport | 9.80 | 2.78 | . | . | 11.57 | 2.70 | 12.00 | . |
| Public administration | 8.73 | 1.62 | 8.86 | 1.46 | 10.50 | 2.12 | 10.00 | 2.83 |
| Education | 8.07 | 1.35 | 7.13 | 2.03 | 8.57 | 1.72 | 7.60 | 0.83 |
| Health | 9.00 | 3.00 | 8.20 | 1.10 | 10.00 | 1.63 | 8.00 | 0.00 |
| Other services | 7.55 | 2.42 | 9.17 | 4.09 | 9.33 | 2.60 | 8.25 | 1.76 |
| Observations | 185 |  | 110 |  | 100 |  | 49 |  |

Note: Based on household survey data. Only individuals engaged in wage employment are included.

**Table F. Average working hours per week by sector**

|  | Kenya | | Namibia | | Tanzania | | Zambia | |
| --- | --- | --- | --- | --- | --- | --- | --- | --- |
|  | Mean | SD | Mean | SD | Mean | SD | Mean | SD |
| Agriculture | 26.93 | 13.01 | 51.36 | 17.69 | 36.47 | 17.29 | 38.80 | 19.32 |
| Industry | 44.07 | 22.47 | 63.29 | 36.53 | 60.00 | 20.63 | 59.00 | 19.47 |
| Construction | 33.20 | 11.10 | 61.00 | 12.68 | 61.71 | 17.90 | 45.33 | 4.62 |
| Commerce | 55.45 | 13.48 | 59.78 | 24.77 | 63.00 | . | 41.00 | 1.41 |
| Hospitality | 65.00 | 48.82 | 59.28 | 18.60 | 58.50 | 26.10 | 48.00 | 0.00 |
| Transport | 55.90 | 21.02 | . | . | 77.00 | 22.85 | 60.00 | . |
| Public administration | 48.00 | 17.80 | 46.71 | 18.70 | 69.00 | 21.21 | 62.00 | 31.11 |
| Education | 41.54 | 10.95 | 36.07 | 10.96 | 42.86 | 8.59 | 38.00 | 4.14 |
| Health | 52.40 | 18.99 | 41.00 | 5.48 | 48.00 | 18.83 | 32.00 | 11.31 |
| Other services | 44.00 | 19.13 | 56.50 | 30.79 | 55.22 | 27.19 | 46.33 | 17.30 |
| Observations | 185 |  | 110 |  | 100 |  | 49 |  |

Note: Based on household survey data. Only individuals engaged in wage employment are included.

**Table G. Hourly wages by sector in local currencies**

|  | Kenya (KSH) | | Namibia (NAD) | | Tanzania (TZS) | | Zambia (ZMW) | |
| --- | --- | --- | --- | --- | --- | --- | --- | --- |
|  | Mean | SD | Mean | SD | Mean | SD | Mean | SD |
| Agriculture | 48.98 | 14.31 | 8.33 | 6.14 | 1,147.89 | 868.50 | 8.71 | 10.25 |
| Industry | 63.04 | 67.05 | 9.85 | 15.84 | 555.99 | 398.06 | 4.43 | 1.14 |
| Construction | 63.21 | 5.14 | 11.11 | 7.59 | 1,202.21 | 794.86 | 3.72 | 2.58 |
| Commerce | 39.18 | 21.54 | 7.35 | 7.74 | 1,111.11 | . | 5.71 | 1.35 |
| Hospitality | 80.28 | 65.71 | 15.95 | 12.41 | 482.68 | 285.39 | 4.95 | 4.05 |
| Transport | 97.55 | 50.82 | . | . | 1,048.98 | 893.81 | 18.75 | . |
| Public administration | 158.94 | 72.41 | 17.95 | 11.44 | 1,268.60 | 1573.10 | 16.82 | 2.74 |
| Education | 138.07 | 72.84 | 63.31 | 22.10 | 2,019.05 | 1,183.42 | 43.44 | 6.67 |
| Health | 131.74 | 82.71 | 32.27 | 24.08 | 2,333.33 | 1,541.10 | 4.69 | 2.21 |
| Other services | 65.28 | 58.47 | 9.36 | 8.04 | 870.87 | 971.25 | 8.74 | 13.41 |
| Observations | 185 |  | 110 |  | 100 |  | 49 |  |

Note: Based on household survey data. Only individuals engaged in wage employment are included.

**Table H. Hourly wages by sector in PPP dollars**

|  | Kenya | | Namibia | | Tanzania | | Zambia | |
| --- | --- | --- | --- | --- | --- | --- | --- | --- |
|  | Mean | SD | Mean | SD | Mean | SD | Mean | SD |
| Agriculture | 1.14 | 0.33 | 1.16 | 0.86 | 1.29 | 0.98 | 1.35 | 1.58 |
| Industry | 1.47 | 1.56 | 1.37 | 2.21 | 0.63 | 0.45 | 0.68 | 0.18 |
| Construction | 1.47 | 0.12 | 1.55 | 1.06 | 1.36 | 0.90 | 0.57 | 0.40 |
| Commerce | 0.91 | 0.50 | 1.03 | 1.08 | 1.25 | . | 0.88 | 0.21 |
| Hospitality | 1.87 | 1.53 | 2.23 | 1.73 | 0.54 | 0.32 | 0.76 | 0.63 |
| Transport | 2.27 | 1.18 | . | . | 1.18 | 1.01 | 2.90 | . |
| Public administration | 3.70 | 1.69 | 2.50 | 1.60 | 1.43 | 1.77 | 2.60 | 0.42 |
| Education | 3.22 | 1.70 | 8.83 | 3.08 | 2.28 | 1.33 | 6.71 | 1.03 |
| Health | 3.07 | 1.93 | 4.50 | 3.36 | 2.63 | 1.74 | 0.72 | 0.34 |
| Other services | 1.52 | 1.36 | 1.31 | 1.12 | 0.98 | 1.10 | 1.35 | 2.07 |
| Observations | 185 |  | 110 |  | 100 |  | 49 |  |

Note: Based on household survey data. Only individuals engaged in wage employment are included.

**3. Robustness checks**

**Table I. Factors influencing individual labor conditions: pooled samples from Tanzania–Kenya and Namibia–Zambia**

|  | (1) | (2) | (3) | (4) |
| --- | --- | --- | --- | --- |
|  | Tanzania & Kenya | | Namibia & Zambia | |
|  | Hourly wage | Decent work | Hourly wage | Decent work |
|  | (PPP ($)) | index (DWI) | (PPP ($)) | index (DWI) |
| Sex (1 = male) | -0.031 | -0.008 | 0.436 | -0.034 |
|  | (0.134) | (0.020) | (0.327) | (0.023) |
| Age (years) | 0.002 | 0.002** | -0.008 | 0.003*** |
|  | (0.006) | (0.001) | (0.011) | (0.001) |
| Level of education (years) | 0.032 | 0.002 | 0.042 | 0.004 |
|  | (0.022) | (0.003) | (0.039) | (0.004) |
| Experience(years) | 0.025*** | 0.000 | 0.020 | 0.001 |
|  | (0.010) | (0.001) | (0.021) | (0.002) |
| In job training (1 = yes) | 1.046*** | 0.234*** | 0.562 | 0.081*** |
|  | (0.222) | (0.032) | (0.391) | (0.027) |
| *Employment location in rural as a reference* | | | | |
| Town within the district/region | 0.777*** | 0.081** | -0.175 | 0.036 |
|  | (0.261) | (0.041) | (0.528) | (0.030) |
| Town outside the district/region | 0.693*** | 0.096** | 0.161 | 0.019 |
|  | (0.230) | (0.038) | (0.288) | (0.037) |
| *Sector dummies (Agriculture as a reference)* | | | | |
| Industry | -0.682*** | 0.003 | -0.566 | -0.016 |
|  | (0.211) | (0.049) | (0.445) | (0.055) |
| Construction | -0.218 | -0.028 | -0.132 | 0.009 |
|  | (0.237) | (0.037) | (0.538) | (0.049) |
| Commerce | -0.937*** | -0.178** | -0.004 | 0.049 |
|  | (0.290) | (0.073) | (0.587) | (0.052) |
| Hospitality | -0.758** | 0.010 | 0.633 | 0.139*** |
|  | (0.318) | (0.056) | (0.449) | (0.053) |
| Transport | -0.302 | -0.055 | 1.420 | 0.139* |
|  | (0.321) | (0.059) | (0.896) | (0.072) |
| Public administration | 0.714* | 0.150*** | 0.789 | 0.279*** |
|  | (0.412) | (0.047) | (0.619) | (0.059) |
| Education | 0.498* | 0.136*** | 6.144*** | 0.359*** |
|  | (0.274) | (0.040) | (0.792) | (0.061) |
| Health | 0.226 | 0.063 | 1.889 | 0.236*** |
|  | (0.561) | (0.069) | (1.174) | (0.081) |
| Other services | -0.311** | 0.005 | 0.262 | 0.097** |
|  | (0.153) | (0.024) | (0.432) | (0.048) |
| Constant | 0.723* | 0.276*** | -0.011 | 0.217*** |
|  | (0.368) | (0.054) | (0.774) | (0.062) |
| Country fixed effects | Yes | Yes | Yes | Yes |
| R-Squared | 0.505 | 0.593 | 0.708 | 0.679 |
| Observations | 285 | 285 | 159 | 159 |

Note: Coefficient estimates from OLS regressions are shown with robust standard errors in parentheses. Columns (1) and (2) pool data from Tanzania and Kenya (East Africa), while columns (3) and (4) pool data from Namibia and Zambia (Southern Africa). The sample comprises individuals engaged in wage employment based on household survey data. * Significant at the 10% level; ** Significant at the 5% level; *** Significant at the 1% level.

**Table J. Factors influencing hourly wages at different quantiles**

|  | (1) | (2) | (3) |
| --- | --- | --- | --- |
|  |  | Quantiles |  |
|  | 0.25 | 0.50 | 0.75 |
| Sex (1 = male) | -0.027 | -0.065 | 0.139 |
|  | (0.083) | (0.060) | (0.112) |
| Age (years) | -0.008*** | -0.007** | -0.002 |
|  | (0.003) | (0.003) | (0.006) |
| Level of education (years) | 0.020** | 0.032*** | 0.034* |
|  | (0.009) | (0.009) | (0.020) |
| Experience (years) | 0.024*** | 0.025*** | 0.020** |
| In job training (1 = yes) | (0.004)  0.328*** | (0.004)  0.572*** | (0.007)  1.437*** |
|  | (0.121) | (0.181) | (0.345) |
| *Employment location in rural as a reference* |  |  |  |
| Town within the district/region | 0.112* | 0.047 | 0.193 |
|  | (0.062) | (0.090) | (0.514) |
| Town outside the district/region | 0.091 | 0.270* | 0.160 |
|  | (0.102) | (0.158) | (0.379) |
| *Sector dummies (Agriculture as a reference)* |  |  |  |
| Industry | -0.390*** | -0.636*** | -0.462** |
|  | (0.107) | (0.092) | (0.221) |
| Construction | -0.010 | 0.004 | -0.099 |
|  | (0.149) | (0.110) | (0.148) |
| Commerce | -0.522*** | -0.712*** | -0.235 |
|  | (0.138) | (0.102) | (0.565) |
| Hospitality | -0.000 | -0.326 | -0.373 |
|  | (0.153) | (0.207) | (0.662) |
| Transport | 0.077 | 0.397 | 0.474 |
|  | (0.179) | (0.399) | (0.767) |
| Public administration | 0.494 | 0.445 | 1.950*** |
|  | (0.344) | (0.772) | (0.305) |
| Education | 1.549** | 2.522*** | 3.653*** |
|  | (0.658) | (0.259) | (0.784) |
| Health | 0.192 | 0.768 | 1.158* |
|  | (0.234) | (1.295) | (0.668) |
| Other services | -0.196* | -0.313*** | -0.012 |
|  | (0.108) | (0.074) | (0.140) |
| Constant | 0.844*** | 0.944*** | 0.847*** |
|  | (0.163) | (0.147) | (0.300) |
| Country fixed effects | Yes | Yes | Yes |
| Observations | 444 | 444 | 444 |

Note: Coefficient estimates from quantile regressions are shown with robust standard errors in parentheses. Pooled data from the household survey across the four study countries, only including individuals engaged in wage employment. * Significant at the 10% level; ** Significant at the 5% level; *** Significant at the 1% level.

**Table K. Factors associated with decent work incidence and decent work index across quantiles**

|  | (1) | (2) | (3) | (4) |
| --- | --- | --- | --- | --- |
|  | DW |  | Quantiles |  |
|  | Incidence | 0.25 | 0.50 | 0.75 |
| Sex (1 = male) | 0.007 | -0.011 | -0.015 | -0.004 |
| Age (years) | (0.032)  0.005*** | (0.019)  0.003*** | (0.018)  0.003*** | (0.010)  0.001** |
|  | (0.001) | (0.001) | (0.001) | (0.001) |
| Level of education (years) | -0.003 | 0.003 | 0.006** | 0.002 |
|  | (0.005) | (0.003) | (0.003) | (0.002) |
| Experience (years) | -0.005** | 0.001 | -0.001 | -0.000 |
| In job training (1 = yes) | (0.002)  0.148*** | (0.001)  0.146*** | (0.001)  0.130*** | (0.001)  0.192*** |
|  | (0.033) | (0.020) | (0.020) | (0.024) |
| *Employment location in rural as a reference* |  |  |  |  |
| Town within the district/region | 0.070* | 0.064* | 0.082** | 0.074*** |
|  | (0.042) | (0.034) | (0.033) | (0.027) |
| Town outside the district/region | -0.031 | 0.014 | 0.032 | 0.092** |
|  | (0.054) | (0.024) | (0.042) | (0.039) |
| *Sector dummies (Agriculture as a reference)* |  |  |  |  |
| Industry | -0.001 | -0.039 | 0.014 | 0.003 |
|  | (0.089) | (0.041) | (0.061) | (0.055) |
| Construction | 0.125* | 0.004 | -0.023 | -0.058 |
|  | (0.075) | (0.054) | (0.024) | (0.037) |
| Commerce | -0.059 | -0.026 | -0.091** | -0.069* |
|  | (0.105) | (0.087) | (0.041) | (0.042) |
| Hospitality | 0.153*** | 0.038 | 0.115** | 0.109*** |
|  | (0.053) | (0.038) | (0.053) | (0.030) |
| Transport | -0.016 | -0.041 | -0.013 | 0.064 |
| Public administration | (0.109)  0.157*** | (0.111)  0.262*** | (0.056)  0.220*** | (0.055)  0.181*** |
| Education | (0.056)  0.126* | (0.028)  0.235*** | (0.027)  0.268*** | (0.050)  0.240*** |
| Health | (0.065)  0.119** | (0.047)  0.179** | (0.033)  0.150** | (0.027)  0.163** |
|  | (0.056) | (0.069) | (0.063) | (0.064) |
| Other services | 0.049 | 0.028 | -0.004 | 0.048** |
| Constant | (0.062)  0.576*** | (0.023)  0.147*** | (0.025)  0.207*** | (0.023)  0.385*** |
|  | (0.090) | (0.044) | (0.043) | (0.029) |
| Country fixed effects | Yes | Yes | Yes | Yes |
| R-Squared | 0.172 |  |  |  |
| Observations | 444 | 444 | 444 | 444 |

Note: Column (1) reports coefficient estimates from an OLS regression of decent work incidence. Columns (2)-(4) report coefficient estimates from quantile regressions at the 25th, 50th, and 75th percentiles of the decent work distribution. Robust standard errors in parentheses. Pooled data from the household survey across the four study countries, only including individuals engaged in wage employment. * Significant at the 10% level; ** Significant at the 5% level; *** Significant at the 1% level.

**Table L. Factors associated with decent work indicators**

|  | (1) | (2) | (3) | (4) | (5) |
| --- | --- | --- | --- | --- | --- |
|  | Adequate | Decent | Job | Safe work | Social |
|  | earning | working time | stability | environment | security |
| Sex (1 = male) | 0.029* | -0.023 | -0.003 | -0.042 | -0.007 |
|  | (0.017) | (0.026) | (0.031) | (0.031) | (0.030) |
| Age (years) | 0.001 | -0.000 | 0.004*** | 0.002 | 0.004*** |
|  | (0.001) | (0.001) | (0.001) | (0.001) | (0.001) |
| Level of education (years) | 0.003 | 0.005 | 0.010* | -0.005 | 0.006 |
|  | (0.003) | (0.004) | (0.006) | (0.005) | (0.005) |
| Experience (years) | 0.002 | 0.005*** | 0.001 | -0.007*** | 0.002 |
|  | (0.001) | (0.002) | (0.002) | (0.002) | (0.002) |
| In job training (1 = yes) | 0.421*** | 0.043 | 0.134*** | -0.041 | 0.264*** |
|  | (0.023) | (0.039) | (0.042) | (0.039) | (0.045) |
| *Employment location in rural as a reference* | | | | | |
| Town within the district/region | 0.056** | 0.075* | 0.060 | -0.012 | 0.078* |
|  | (0.028) | (0.044) | (0.049) | (0.055) | (0.047) |
| Town outside the district/region | 0.120*** | 0.036 | -0.068 | 0.085* | 0.117** |
|  | (0.028) | (0.048) | (0.056) | (0.051) | (0.049) |
| *Sector dummies (Agriculture as a reference)* | | | | | |
| Industry | -0.060* | -0.152** | 0.055 | -0.055 | 0.138** |
|  | (0.030) | (0.063) | (0.077) | (0.076) | (0.057) |
| Construction | -0.042 | -0.072 | -0.030 | 0.119* | -0.078* |
|  | (0.039) | (0.058) | (0.069) | (0.069) | (0.043) |
| Commerce | -0.008 | -0.286*** | -0.077 | -0.009 | 0.044 |
|  | (0.038) | (0.069) | (0.090) | (0.079) | (0.067) |
| Hospitality | 0.012 | -0.071 | 0.133* | 0.118** | 0.187*** |
|  | (0.032) | (0.057) | (0.069) | (0.051) | (0.065) |
| Transport | -0.007 | -0.219*** | 0.041 | 0.069 | 0.108 |
| Public administration | (0.050)  0.083** | (0.081)  0.148** | (0.085)  0.273*** | (0.084)  0.178** | (0.097)  0.370*** |
| Education | (0.042)  0.130*** | (0.073)  0.179*** | (0.052)  0.155** | (0.073)  0.211*** | (0.072)  0.420*** |
|  | (0.034) | (0.055) | (0.064) | (0.062) | (0.058) |
| Health | 0.054 | 0.080 | 0.152* | 0.157** | 0.292*** |
|  | (0.055) | (0.073) | (0.086) | (0.072) | (0.093) |
| Other services | 0.013 | -0.008 | 0.051 | 0.033 | 0.117*** |
|  | (0.023) | (0.039) | (0.047) | (0.051) | (0.039) |
| Constant | -0.015 | 0.457*** | 0.198** | 0.709*** | -0.147* |
|  | (0.041) | (0.065) | (0.083) | (0.083) | (0.076) |
| Country fixed effects | Yes | Yes | Yes | Yes | Yes |
| R-Squared | 0.727 | 0.280 | 0.314 | 0.156 | 0.572 |
| Observations | 444 | 444 | 444 | 444 | 444 |

Note: Coefficient estimates from OLS regressions are shown with robust standard errors in parentheses. Each column represents a separate regression with a different dimension of decent work as the dependent variable. Pooled data from the household survey across the four study countries, only including individuals engaged in wage employment. * Significant at the 10% level; ** Significant at the 5% level; *** Significant at the 1% level.

**References**

1. Nunnally JC. Psychometric theory. 2nd ed. New York: McGraw-Hill; 1978.

2. Lance CE, Butts MM, Michels LC. The sources of four commonly reported cutoff criteria: What did they really say? Organ Res Methods. 2006;9: 202–220. doi:10.1177/1094428105284919

3. Cortina JM. What is coefficient alpha? An examination of theory and applications. J Appl Psychol. 1993;78: 98–104. doi:10.1037/0021-9010.78.1.98

4. Nunnally JC. Psychometric theory. New York: McGraw-Hill; 1967.

5. Nunnally JC, Hernstein IH. Psychometric theory. 3rd ed. New York: McGraw-Hill; 1994.

6. Streiner DL. Starting at the beginning: An introduction to coefficient alpha and internal consistency. J Pers Assess. 2003;80: 99–103. doi:10.1207/S15327752JPA8001_18

7. Tavakol M, Dennick R. Making sense of Cronbach’s alpha. Int J Med Educ. 2011;2: 53–55. doi:10.5116/ijme.4dfb.8dfd
